# TIMP-2 mediates the anti-invasive effects of the nitric oxide-releasing prodrug JS-K in breast cancer cells

**DOI:** 10.1186/bcr2095

**Published:** 2008-05-12

**Authors:** Ann-Marie Simeone, Vanity McMurtry, René Nieves-Alicea, Joseph E Saavedra, Larry K Keefer, Marcella M Johnson, Ana M Tari

**Affiliations:** 1 Department of Experimental Therapeutics, The University of Texas MD Anderson Cancer Center, Houston, TX 77030, USA; 2 Basic Research Program, SAIC-Frederick, National Cancer Institute at Frederick, Frederick, MD 21702, USA; 3 Laboratory of Comparative Carcinogenesis, National Cancer Institute at Frederick, Frederick, MD 21702, USA; 4 Department of Biostatistics & Applied Mathematics, The University of Texas MD Anderson Cancer Center, Houston, TX 77030, USA

## Abstract

**Introduction:**

Tumor invasion and metastasis remain a major cause of mortality in breast cancer patients. High concentrations of nitric oxide (NO) suppress tumor invasion and metastasis *in vivo*. NO prodrugs generate large amounts of NO upon metabolism by appropriate intracellular enzymes, and therefore could have potential in the prevention and therapy of metastatic breast cancer.

**Methods:**

The present study was designed to determine the effects of the NO-releasing prodrug O^2^-(2,4-dinitrophenyl) 1- [(4-ethoxycarbonyl)piperazin-1-yl]diazen-1-ium-1,2-diolate (JS-K) on breast cancer invasion and the mechanisms involved. MDA-MB-231, MDA-MB-231/F10, and MCF-7/COX-2 were the three breast cancer cell lines tested. NO levels were determined spectrophotometrically using a NO assay kit. Invasion and the expression of matrix metalloproteinases (MMPs) and tissue inhibitor of MMPs were determined using Matrigel invasion assays, an MMP array kit and ELISAs. The activity and expression of extracellular signal-regulated kinase 1/2, p38, and c-Jun N-terminal kinase mitogen-activated protein kinases were determined using western blot analyses.

**Results:**

Under conditions by which JS-K was not cytotoxic, JS-K significantly decreased (*P *< 0.05) the invasiveness of breast cancer cells across the Matrigel basement membrane, which was directly correlated with NO production. JS-43-126, a non-NO-releasing analog of JS-K, had no effect on NO levels or invasion. JS-K increased (*P *< 0.05) TIMP-2 production, and blocking TIMP-2 activity with a neutralizing antibody significantly increased (*P *< 0.05) the invasive activity of JS-K-treated cells across Matrigel. JS-K decreased p38 activity, whereas the activity and the expression of extracellular signal-regulated kinase 1/2 and c-Jun N-terminal kinase were unaffected.

**Conclusion:**

We report the novel findings that JS-K inhibits breast cancer invasion across the Matrigel basement membrane, and NO production is vital for this activity. Upregulation of TIMP-2 production is one mechanism by which JS-K mediates its anti-invasive effects. JS-K and other NO prodrugs may represent an innovative biological approach in the prevention and treatment of metastatic breast cancer.

## Introduction

Breast cancer is the most common cancer detected in women, accounting for nearly one out of every three cancers diagnosed in the United States. Metastasis is the primary cause of breast cancer mortality. The 5-year survival rate for women diagnosed with localized breast cancer is 98%, which contrasts dramatically with the 27% survival rate of women diagnosed with distant metastasis breast cancer [1] (data based on the November 2006 SEER data submission, posted to the SEER Okay web site in 2007. Development of effective chemopreventive and therapeutic strategies for metastatic disease is urgently needed.

The free radical nitric oxide (NO) plays an important role in regulating tumor growth and metastasis. The amount of NO produced depends on the expression of nitric oxide synthase (NOS) isoforms. NOSI and NOSIII are expressed constitutively and produce trace amounts of NO. NOSII is the inducible isoform and can generate large amounts of NO.

Low concentrations of NOSIII-derived NO promoted the growth, invasion, and metastasis of murine mammary tumors [2,3]. In contrast, high levels of NOSII-mediated NO have been shown to suppress tumorigenesis and metastasis *in vivo *[4-8]. EMT-6J murine breast carcinoma cells, which constitutively expressed inducible NOSII and secreted high levels of NO, had a lower metastatic potential than NOSII-deficient EMT-6H cells when injected into mice [6]. EMT-6H cells induced the formation of numerous metastases in the lungs of all the injected mice, while the number of mice with lung metastases and the number of metastases per lung were lower in the EMT-6J group [6]. Similarly, pancreatic cells transduced with wild-type *NOSII *suppressed tumor growth and distant metastasis to the liver in an orthotopic xenograft model [8].

We previously demonstrated that breast cancer cells possess intrinsic resistance mechanisms that can prevent the induction of NOSII [9,10]; any chemopreventive or therapeutic strategy designed to produce high NO levels in such cells should therefore not depend on NOSII induction. Given the suppressive effects of high levels of NO on tumorigenesis and metastasis, drugs that supply NO exogenously could have potential in breast cancer therapy and chemoprevention. The challenge is to deliver NO in a sustained and controlled manner.

NO donors that spontaneously generate large amounts of NO independent of NOSII induction are activated at physiological pH and can induce NO-mediated systemic hypotension. NO prodrugs are another type of NOSII-independent NO-releasing agent. NO prodrugs do not release NO spontaneously, but rather can be activated to generate high concentrations of NO upon metabolism by intracellular enzyme targets. Arylated diazeniumdiolates have been designed to be activated for NO release by reaction with glutathione S-transferases (GSTs). GSTs are a superfamily of enzymes that detoxify xenobiotics by conjugating them to glutathione and increasing their cellular excretion. Among the major isoforms (α, μ, π), GST-π is expressed at the highest concentration in breast tumors [11,12]. The expression of GST-π is associated with more aggressive tumors, poor prognosis, increased risk of relapse, and decreased disease-free survival in breast cancer patients [13,14].

O^2^-(2,4-dinitrophenyl) 1- [(4-ethoxycarbonyl)piperazin-1-yl]diazen-1-ium-1,2-diolate (JS-K), a diazeniumdiolate activated to release high levels of NO by GST enzymes [15], has been shown to inhibit cancer cell growth *in vitro *and *in vivo *[15-19]. Whether JS-K can suppress cancer invasion, however, is not known. In the present article we report the novel findings that JS-K inhibits the invasive activity of breast cancer cells across the Matrigel basement membrane at doses by which JS-K was not cytotoxic, and that increasing TIMP-2 production is one mechanism by which JS-K mediates its anti-invasive effects. The results presented here have a bearing on the potential for NO prodrugs to be used in the prevention and therapy of metastatic breast cancer.

## Materials and methods

### Reagents

Matrigel and type I collagen were purchased from BD Biosciences (Bedford, MA, USA) and Sigma-Aldrich Chemical Co (St. Louis, MO, USA), respectively. Hema-3 was purchased from Fisher Scientific (Middleton, VA, USA). Rabbit polyclonal GST-π and GST-α antibodies were purchased from EMD Biosciences (La Jolla, CA, USA). β-actin monoclonal antibody was purchased from Sigma-Aldrich Chemical Co. The polyclonal antibody to TIMP-2 was purchased from R & D Systems (Minneapolis, MN, USA). Phospho(Thr^202^/Tyr^204^)-extracellular signal-regulated kinase 1/2 (ERK1/2), ERK1/2, phospho(Thr^180^/Tyr^182^)-p38, p38, phospho(Thr^183^/Tyr^185^)-c-Jun N-terminal kinase (JNK), and JNK antibodies were purchased from Cell Signaling (Beverly, MA, USA).

**Figure 1:**
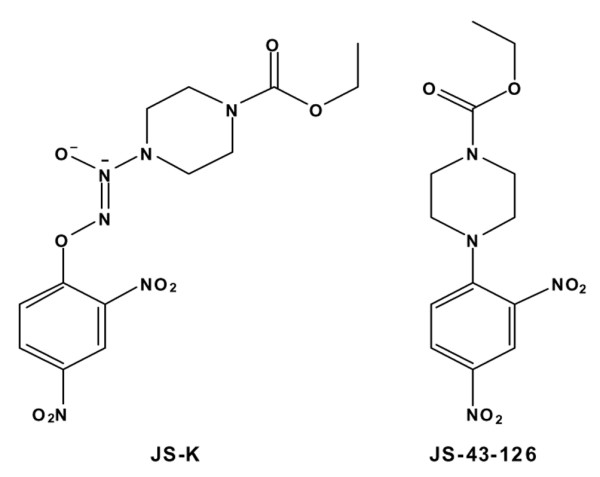
Structures of JS-K and JS-43-126, a non-nitric oxide-releasing JS-K analog.

JS-K [20] and JS-43-126 [21], a JS-K analog that does not release NO, were prepared as previously described. Stock solutions of JS-K and JS-43-126 were prepared in dimethylsulfoxide and were stored at -20°C. The structures of JS-K and JS-43-126 are presented in Figure 1.

### Cell lines and culture conditions

The human MDA-MB-231 breast cancer cell line was obtained from American Type Cell Culture (Manassas, VA, USA). The MDA-MB-231 cell line is an estrogen-independent, highly metastatic human breast cancer cell line. Breast cancer commonly metastasizes to the skeletal system. MDA-MB-231/F10 (F10) is a bone metastatic derivative of MDA-MB-231 cells selected *in vivo *by repeated intracardiac injections of the MDA-MB-231 cells into female nude mice until no micrometastases were detected histologically in any organs other than bone [22]. The F10 cell line was kindly provided by Dr Toshiyuki Yoneda (The University of Texas Health Science Center, San Antonio, TX, USA).

Breast cancer also commonly metastasizes to lymph nodes. Elevated COX-2 expression in invasive breast tumor is associated with lymph node metastasis [23-25]. MCF-7/COX-2 cells are estrogen-dependent MCF-7 cells stably transfected with plasmids encoding the human COX-2 gene [26]. The parental MCF-7 cells are poorly invasive but the MCF-7/COX-2 cells are highly invasive [27].

The MDA-MB-231 and F10 cell lines were cultured in DMEM/F12 (Invitrogen, Carlsbad, CA, USA) supplemented with 5% heat-inactivated FBS) (Invitrogen) at 37°C under 5% carbon dioxide in a humidified incubator. MCF-7/COX-2 cells were continuously cultured in DMEM/F12 medium containing 5% FBS and 500 μg/ml antibiotic G418 (Invitrogen).

### Western blot analysis

Protein lysates (50 μg) from untreated exponentially growing MDA-MB-231, F10, and MCF-7/COX-2 breast cancer cells were loaded onto 15% polyacrylamide gels to determine the expression of GST-π and GST-α. The MDA-MB-231 cells (3 × 10^5 ^cells), F10 cells (3 × 10^5 ^cells), and MCF-7/COX-2 cells (4 × 10^5 ^cells) were plated in T25 flasks in 5 ml DMEM/F12 medium supplemented with 5% FBS. The next day, cells were treated with JS-K (0.5 and 1.0 μM) for 24 hours. Protein lysates (30 μg) were loaded onto 12% polyacrylamide gels to determine the activity and expression of ERK1/2, p38, and JNK mitogen-activated protein kinases. Proteins were electrophoresed and electrotransferred as described previously [10]. Membranes were incubated with the appropriate antibodies. β-actin was used as a loading control. Protein bands were visualized by enhanced chemiluminescence (Kirkgaard & Perry Laboratories, Gaithersburg, MD, USA). Images were scanned and quantified by an Alpha Innotech densitometer using the Alpha Imager application program (San Leandro, CA, USA).

### Nitric oxide assay

The MDA-MB-231 cells (3 × 10^5 ^cells), F10 cells (3 × 10^5 ^cells), and MCF-7/COX-2 cells (4 × 10^5 ^cells) were plated in T25 flasks in 5 ml DMEM/F12 medium supplemented with 5% FBS. The next day, cells were treated with JS-K (0.5 and 1.0 μM) or JS-43-126 (0.5 and 1.0 μM) for 72 hours. The medium was recovered, centrifuged for 5 minutes, and concentrated using spin columns with 10-kDa-cutoff filters (Millipore, Bedford, MA, USA). Total NO was determined in the conditioned concentrated supernatants by quantifying nitrite, the stable end product of NO oxidation, spectrophotometrically using a colorimetric nonenzymatic nitric oxide assay kit (Oxford Biomedical Research, Oxford, MI, USA) as described previously [9]. Cell growth was determined by total live cell counts using trypan blue exclusion. Nitrite values were normalized for total cell counts and expressed as picomoles per 10^6 ^cells. The experiments were performed in triplicate wells.

### Proliferation assay

One hundred microliters of Matrigel (0.7 mg/ml) were added to each well of 96-well plates. The MDA-MB-231 cells, F10 cells, and MCF/COX-2 cells (1 × 10^4 ^cells resuspended in 100 μl medium) were added to Matrigel-coated wells. The next day, JS-K (0, 0.5 and 1.0 μM) was added to cells in pentaplicate wells. After 3 days of incubation, cell proliferation was determined by the Promega (Madison, WI, USA) Celltiter 96^® ^AQ_ueous _nonradioactive cell proliferation assay. The CellTiter 96^® ^AQ_ueous _Assay is composed of 3-(4,5-dimethylthiazol-2-yl)-5-(3-carboxymethoxyphenyl)-2-(4-sulfophenyl)-2H-tetrazolium (MTS) and the electron coupling reagent phenazine methosulfate. MTS is reduced by live cells into a formazan product, whose absorbance can be read at 490 nm. The quantity of formazan product is directly proportional to the number of living cells in culture. The absorbance of the formazan product was read within 2 hours after the MTS/phenazine methosulfate dye addition. Cell proliferation was expressed as the percentage of untreated cells. The experiments were repeated twice.

### Invasion assay

The effect of JS-K on breast cancer invasion was determined *in vitro *using modified Boyden chamber assays as previously described [28]. Briefly, six-well plate transwell inserts with 8-μm pore-size polycarbonate filters (Fisher Scientific) were coated with Matrigel (0.7 mg/ml) or type I collagen (20 μg/ml) in cold serum-free DMEM/F12 medium and were placed at room temperature for 40 minutes. Cells were trypsinized, resuspended in serum-supplemented media, and were then counted.

Cells were then washed three times with serum-free medium. The MDA-MB-231 cells (3 × 10^5 ^cells), F10 cells (3 × 10^5 ^cells), and MCF-7/COX-2 cells (4 × 10^5 ^cells), resuspended in 500 μl, were added into the Matrigel-coated or the collagen-coated transwell inserts and were incubated for 72 hours in the absence or presence of JS-K (0.5 and 1.0 μM) or JS-43-126 (0.5 and 1.0 μM). The lower chambers were filled with 2 ml DMEM/F12 medium supplemented with 5% FBS. After incubation, noninvading cells on the upper surface of the filter were removed with cotton swabs. Cells that had invaded through the pores onto the lower side of the filter were fixed, stained with Hema-3, and photographed. The invaded cells were counted in five fields for each filter under a light microscope at 40× magnification. The invasiveness of the cells was expressed as the mean number of cells that had invaded to the lower side of the filter. The experiments were performed in triplicate wells and were repeated twice.

To determine the importance of TIMP-2 in JS-K-mediated anti-invasive effects, TIMP-2 activity was blocked with a neutralizing antibody (R & D Systems). The MDA-MB-231, F10, and MCF-7/COX-2 cells were treated with 1 μM JS-K in the presence or absence of the anti-TIMP-2 antibody (2.5 μg/ml) for 72 hours in a Matrigel invasion assay. The experiments were performed in triplicate wells and were repeated twice.

### Collection of conditioned medium supernatant

The MDA-MB-231 cells (3 × 10^5 ^cells), F10 cells (3 × 10^5 ^cells), and MCF-7/COX-2 cells (4 × 10^5 ^cells) were plated in T25 flasks in 5 ml DMEM/F12 medium supplemented with 5% FBS. The next day, cells were treated with JS-K (0.5 and 1.0 μM) or JS-43-126 (0.5 and 1.0 μM) for 24 hours. The medium in each flask was then replaced with serum-free medium and the flasks were incubated for an additional 24 hours. The medium was recovered, centrifuged for 5 minutes, and concentrated using spin columns with 10-kDa-cutoff filters. The medium collected was used for the matrix metalloproteinase (MMP) array and to determine the expression of TIMP-2.

### Human matrix metalloproteinase array

The expression of MMPs and TIMPs in the conditioned medium supernatant was qualitatively screened using a human MMP array kit (RayBiotech, Norcross, GA, USA). The array allows for the simultaneous detection of seven MMPs (MMP-1, MMP-2, MMP-3, MMP-8, MMP-9, MMP-10, MMP-13) and three TIMPs (TIMP-1, TIMP-2, TIMP-4). Images were scanned using an Alpha Imager application program.

### Enzyme-linked immunosorbent assays for TIMP-2

The concentration of TIMP-2 in the conditioned medium supernatant was determined using a TIMP-2 ELISA kit (EMD Biosciences). The concentration of TIMP-2 was normalized to the cell number and was expressed as nanograms per milliliter per 10^6 ^cells. The experiments were performed in triplicate wells and were repeated three times.

### Statistical analyses

For statistical analysis of the invasion experiments, the Shapiro-Wilk test was first performed to assess the normality of assumption data. Given that the data were normally distributed, two-sample *t *tests were performed for each of the three cell lines to compare the number of invading cells for the untreated group with the number of invading cells for each dose of JS-K and JS-43-126. The number of invading cells was also compared between the two doses of JS-K and JS-43-126. Two-sample *t *tests were also used to compare the number of invading cells for the group treated with JS-K with the number of invading cells for the group treated with JS-K in the presence of the anti-TIMP-2 antibody for each cell line.

The significance level for each individual comparison (0.05/3 = 0.0167) was adjusted by the Bonferroni method to account for multiple testing within each cell line to achieve an overall significance level of 5%. All analyses were performed using SAS software (SAS Institute, Inc., Cary, NC, USA). The same statistical analyses were used to compare the NO and TIMP-2 levels of untreated cells with those treated with JS-K or JS-43-126 as appropriate.

## Results

### Expression of GST-π and GST-α in breast cancer cell lines

**Figure 2:**
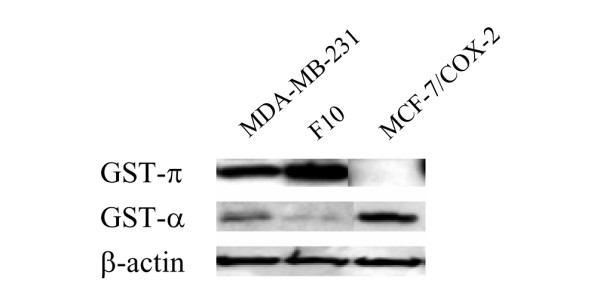
Expression of glutathione S-transferase π and α isoforms in breast cancer cell lines. Expression of glutathione S-transferase (GST)-π and GST-α isoforms in breast cancer cell lines. Protein lysates were obtained from exponentially growing MDA-MB-231, F10, and MCF-7/COX-2 cells. Western blot analyses using polyclonal GST-π and GST-α antibodies were performed. β-actin was used as a loading control.

JS-K is activated to release NO by GST enzymes [15]; the expression of GST-π and GST-α in MDA-MB-231, F10, and MCF-7/COX-2 breast cancer cells was therefore determined. The MDA-MB-231 and F10 cells expressed GST-π and GST-α, but GST-π was the predominant isoform (Figure 2). MCF-7/COX-2 cells expressed GST-α but not GST-π (Figure 2).

### JS-K, but not JS-43-126, increases nitric oxide levels in breast cancer cells

**Figure 3:**
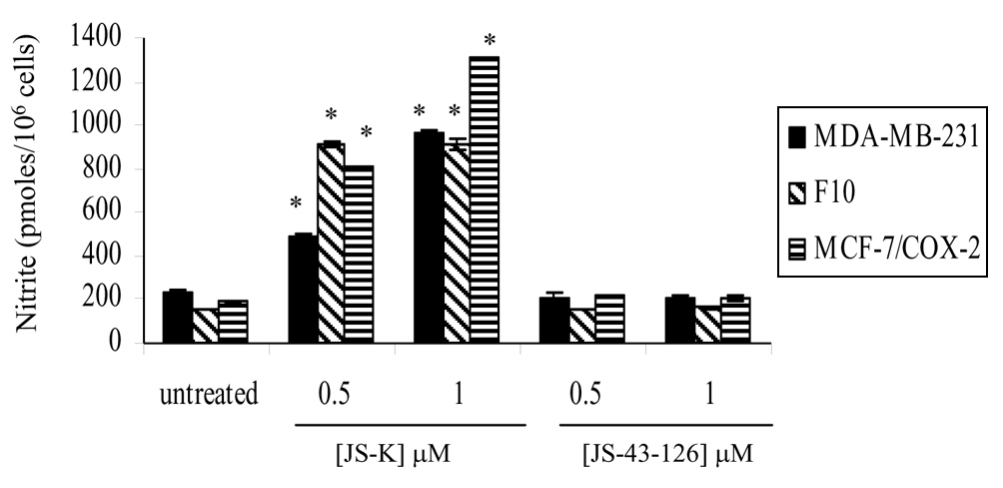
JS-K, but not JS-43-126, increases nitric oxide production in breast cancer cells. Conditioned medium supernatant was collected from MDA-MB-231, F10, and MCF-7/COX-2 cells treated in the absence or presence of JS-K or JS-43-126 for 72 hours. Total nitric oxide (NO) was determined by quantifying nitrite, the stable end product of NO oxidation, spectrophotometrically using a colorimetric nonenzymatic nitric oxide assay kit. Nitrite values were normalized for total cell counts and expressed as picomoles per 10^6 ^cells. Columns indicate the mean of triplicate wells ± standard deviation. *Significant increase in NO levels relative to untreated cells, *P *< 0.05.

NO levels were determined in untreated and JS-K-treated MDA-MB-231, F10, and MCF-7/COX-2 cells to confirm drug activation. The NO production was significantly increased (*P *< 0.05) in the three cell lines as a result of JS-K treatment (Figure 3).

The NO levels were 2.1-fold and fourfold higher (*P *< 0.05) in MDA-MB-231 cells treated with 0.5 and 1 μM JS-K, respectively (Figure 3). The NO levels were increased (*P *< 0.05) 5.8-fold and 6.1-fold at the 0.5 and 1 μM concentrations of JS-K in F10 cells, respectively (Figure 3). Although the two concentrations of JS-K did not differ (*P *> 0.05) in the NO levels produced, the NO levels of JS-K-treated F10 cells were significantly higher (*P *< 0.05) in comparison with untreated cells (Figure 3). The NO levels were increased (*P *< 0.05) 4.9-fold and sevenfold in MCF-7/COX-2 cells at the 0.5 and 1 μM concentrations of JS-K, respectively (Figure 3). JS-K can therefore be activated to release NO by breast cancer cells. In contrast, NO production was not different (*P *> 0.05) between untreated cells and those treated with JS-43-126 for each of the three cell lines (Figure 3).

### JS-K, but not JS-43-126, decreases breast cancer invasion across a Matrigel-coated membrane

The invasion of cancer cells through basement membranes is an essential step in cancer metastasis. Matrigel is a solubilized basement membrane preparation extracted from the Engelbreth-Holm-Swarm mouse sarcoma, a tumor rich in extracellular matrix proteins. The major component of Matrigel is laminin. Matrigel has been used by numerous groups to assay the invasive activity of tumor cells across the basement membrane [29-31].

**Figure 4:**
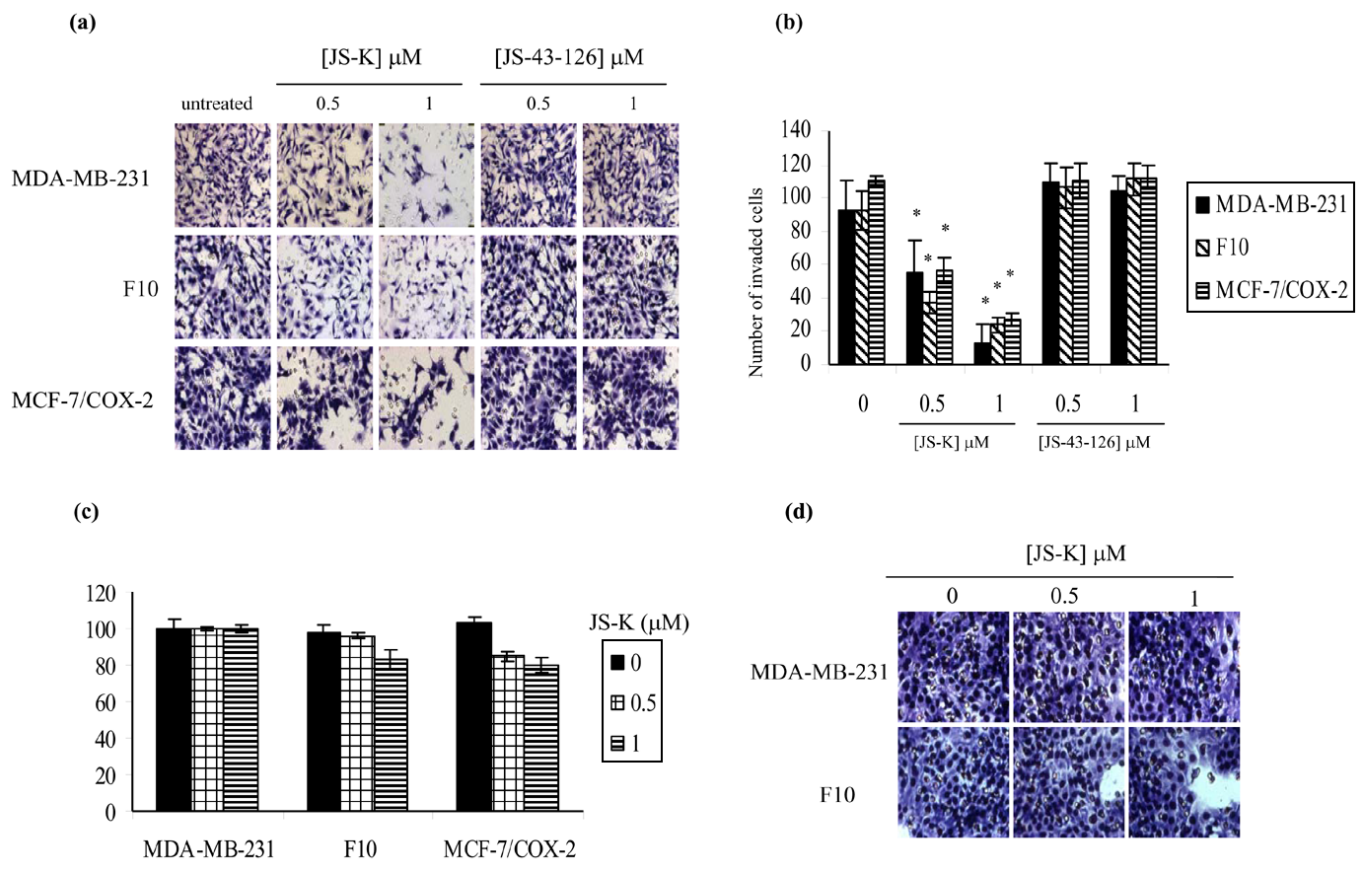
JS-K, but not JS-43-126, decreases the invasiveness of breast cancer cells across Matrigel. **(a) **MDA-MB-231, F10, and MCF-7/COX-2 cells were added into Matrigel-coated transwell inserts and incubated with JS-K or JS-43-126 for 72 hours. Cells that invaded through the pores onto the lower side of the filter were fixed, stained, and photographed. **(b) **The number of invaded cells for each filter was counted in five fields. The invasiveness of the cells is expressed as the mean number of cells that invaded to the lower side of the filter. Columns indicate the mean of triplicate wells ± standard deviation. *Significant decrease in the number of invaded cells relative to untreated cells, *P *< 0.05. **(c) **MDA-MB-231, F10, and MCF-7/COX-2 cells were plated on Matrigel-coated wells and incubated with JS-K. After 72 hours, cell proliferation was determined by Celltiter 96^® ^AQ_ueous _nonradioactive cell proliferation assay. Columns indicate the mean of pentaplicate wells ± standard deviation. **(d) **MDA-MB-231 and F10 cells were added into type I collagen-coated transwell inserts and incubated with JS-K for 72 hours. Cells that invaded through the pores onto the lower side of the filter were fixed, stained, and photographed.

Matrigel invasion assays were performed to determine the effect of JS-K on the invasiveness of breast cancer cells across the basement membrane. Untreated MDA-MB-231, F10, and MCF-7/COX-2 cells displayed a high invasive capacity on Matrigel (Figure 4a). In all cell lines, JS-K significantly (*P *< 0.05) reduced the number of invasive cells (Figure 4a, b). The number of invaded MDA-MB-231 cells was decreased (*P *< 0.05) 37% and 85% at the 0.5 and 1 μM doses of JS-K, respectively (Figure 4b). The number of invaded F10 cells was reduced (*P *< 0.05) 63% and 76% by the 0.5 and 1 μM doses of JS-K, respectively (Figure 4b). The two doses of JS-K, however, did not have significantly different (*P *> 0.05) anti-invasive effects in F10 cells (Figure 4b). In MCF-7/COX-2 cells, JS-K reduced (*P *< 0.05) the number of invaded cells 49% and 75% at the 0.5 and 1 μM doses of JS-K, respectively (Figure 4b). In contrast, the invasiveness of the three cell lines was unaffected by treatment with JS-43-126 (Figure 4b). JS-K can therefore decrease breast cancer invasion across Matrigel, and this is dependent on NO production.

JS-K has been shown to induce growth inhibition in cancer cells [15-19]. We determined the effects of JS-K on the proliferation of breast cancer cells grown on Matrigel, in order to mimic the conditions used in the Matrigel invasion assays. The 0.5 and 1.0 μM doses of JS-K induced < 20% growth inhibition in any of the breast cancer cell lines (Figure 4c). JS-K-mediated decreases in the Matrigel invasion assays were therefore not the result of growth inhibition.

Bone is the most prevalent site of first distant relapse of breast cancer, with as many as 85% of patients with advanced breast cancer suffering from bone metastases [32]. Type I collagen is the most abundant protein within the bone, making up > 90% of the total protein within this site [33]. Type I collagen has been used to assay the invasive activity of tumor cells across the bone matrix [34,35]. A type I collagen invasion assay was performed to determine whether JS-K may inhibit the invasiveness of breast cancer cells across the bone matrix. The conditions for the collagen invasion assay were identical to those of the Matrigel invasion assay, except that type I collagen was used to coat the transwell insert. The MDA-MB-231 and F10 cells displayed a high invasive capacity on type I collagen (Figure 4d), but MCF-7/COX-2 cells did not (data not shown). JS-K did not reduce the invasiveness of breast cancer cells across type I collagen-coated insert (Figure 4d). These data indicate that JS-K can block breast cancer cells from invading through Matrigel but not through type I collagen, suggesting that JS-K can block breast cancer invasion through the basement membrane but not through the bone matrix.

### JS-K increases TIMP-2 production to block breast cancer cells from invading through Matrigel

**Figure 5:**
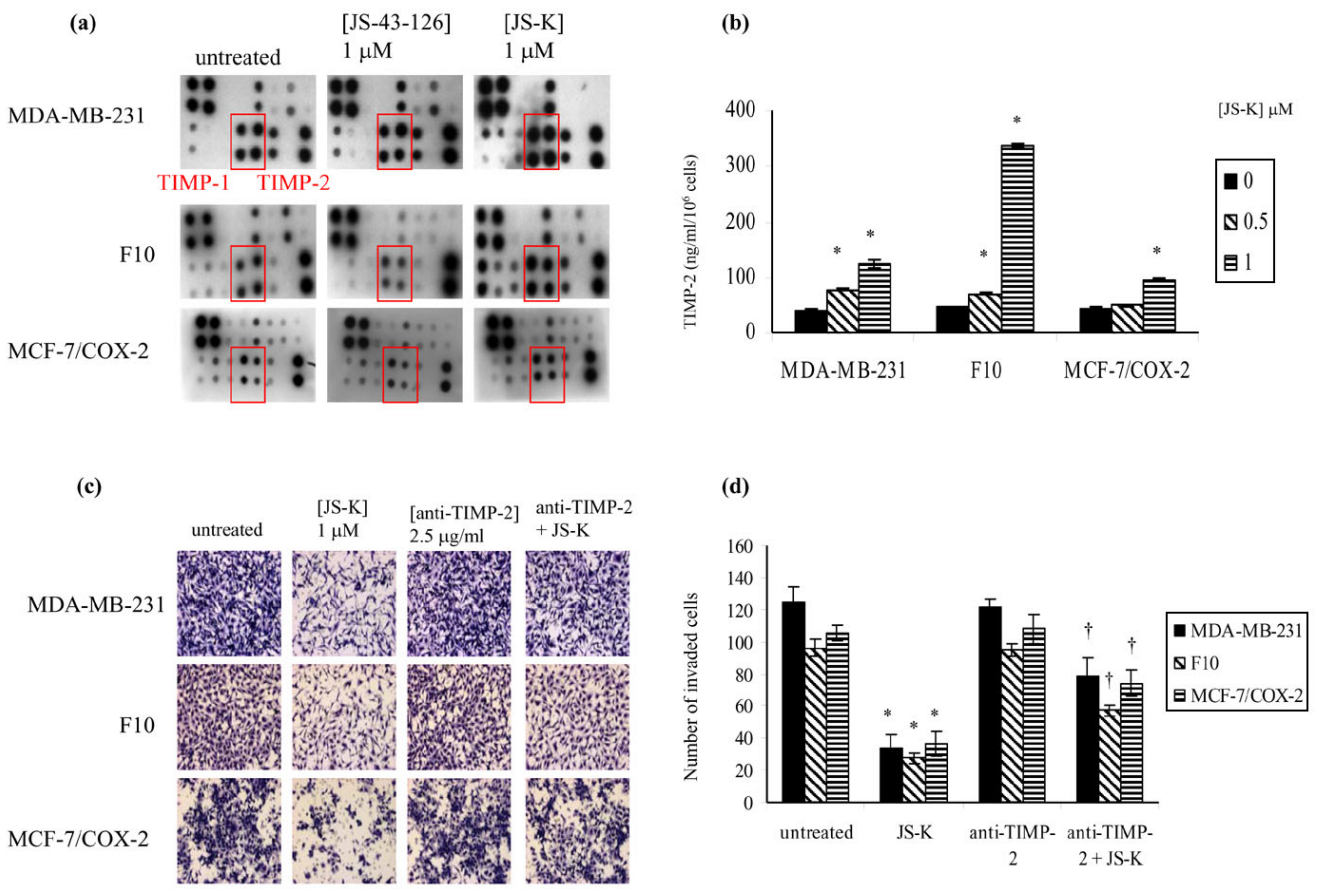
JS-K increases TIMP-2 production to suppress breast cancer invasion through Matrigel. **(a) **Expression of matrix metalloproteinases (MMPs) and tissue inhibitor of matrix metalloproteinases (TIMPs) in the conditioned medium supernatant obtained from untreated and JS-K-treated cells was qualitatively screened using a human MMP array kit. **(b) **TIMP-2 levels in the conditioned medium supernatant obtained from untreated and JS-K-treated cells were determined using a commercial ELISA kit. Columns indicate the mean of triplicate wells ± standard deviation. *Significant increase in the TIMP-2 levels relative to untreated cells, *P *< 0.05. **(c) **MDA-MB-231, F10, and MCF-7/COX-2 cells were treated with JS-K (1 μM) in the presence or absence of a TIMP-2 neutralizing antibody (2.5 μg/ml) for 72 hours in a Matrigel invasion assay. **(d) **The number of invaded cells was counted. Columns indicate the mean of triplicate wells ± standard deviation. *Significant decrease in the number of invaded cells relative to untreated cells, *P *< 0.05. ^†^Increase relative to JS-K-treated cells, *P *< 0.05.

MMPs, which are involved in the degradation of the basement membrane, are essential to the invasive process. In contrast, TIMPs regulate the activity of MMPs and protect the basement membrane from proteolysis. A human MMP array was performed to screen the effects of JS-K on MMP and TIMP production. The array profiles for JS-43-126-treated cells were similar to those of untreated cells (Figure 5a). In contrast, the most consistent effect observed in the arrays of the three cell lines as a result of JS-K treatment was an increase in the production of TIMP-2 (Figure 5a). To confirm the JS-K-mediated increase in TIMP-2 levels that were observed in the MMP arrays, TIMP-2 ELISAs were performed. In MDA-MB-231 cells, TIMP-2 levels were increased (*P *< 0.05) 1.9-fold and threefold at the 0.5 and 1 μM doses of JS-K, respectively, while TIMP-2 was increased (*P *< 0.05) 1.5-fold and 7.2-fold in F10 cells at the same doses (Figure 5b). In MCF-7/COX-2 cells, TIMP-2 was increased (*P *< 0.05) only at the higher dose of JS-K (Figure 5b). TIMP-2 was increased (*P *< 0.05) twofold in MCF-7/COX-2 cells at the 1 μM concentration of JS-K (Figure 5b). These data indicate that TIMP-2 may be the major, but not the only, target of JS-K.

Next we determined the importance of TIMP-2 in the anti-invasive effects of JS-K. To do this, TIMP-2 activity was blocked with a commercially available neutralizing antibody and the effect of JS-K on the invasiveness of MDA-MB-231, F10, and MCF-7/COX-2 cells across Matrigel was determined. At the concentration used, the anti-TIMP-2 antibody had no effect on invasion (Figure 5c, d). JS-K decreased the invasiveness of all cell lines across Matrigel; however, blocking TIMP-2 activity significantly (*P *< 0.05) suppressed the anti-invasive effects of JS-K (Figure 5c, d). In comparison with untreated MDA-MB-231 cells, JS-K decreased (*P *< 0.05) the number of invaded cells by 72% and 37% in the absence and presence of the anti-TIMP-2 antibody, respectively (Figure 5d). The number of invaded F10 cells was 72% and 40% lower (*P *< 0.05) relative to untreated cells when treated with JS-K alone or in combination with the anti-TIMP-2 antibody, respectively (Figure 5d). JS-K decreased (*P *< 0.05) the number of invaded MCF-7/COX-2 cells by 65%, but in the presence of anti-TIMP-2 the number of invaded cells was decreased (*P *< 0.05) by 30% (Figure 5d). These data indicate that TIMP-2 is an important mediator of the anti-invasive activity of JS-K across the Matrigel basement membrane.

### JS-K decreases p38 activity in breast cancer cells

**Figure 6:**
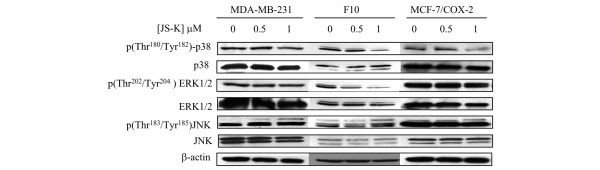
JS-K decreases p38 phosphorylation in breast cancer cells. Protein lysates were obtained from MDA-MB-231, F10, and MCF-7/COX-2 cells treated in the absence or presence of JS-K for 24 hours. Western blot analyses using phospho(Thr^180^/Tyr^182^)-p38, p38, phospho(Thr^202^/Tyr^204^)-extracellular signal-regulated kinase 1/2 (ERK1/2), ERK1/2, phospho(Thr^183^/Tyr^185^)-c-Jun N-terminal kinase (JNK), and JNK antibodies were performed. β-actin was used as a loading control. Images were scanned and quantified. Ratios of phospho-p38 to total p38 levels in JS-K-treated cells were determined and compared with those in untreated cells. Ratios of phospho-ERK1/2 to total ERK1/2 levels in JS-K-treated F10 cells were determined and compared with those in untreated F10 cells.

Mitogen-activated protein kinase pathways, which have been shown to regulate TIMP-2 [36-38], are activated by JS-K [16,17]. We therefore determined whether these pathways were involved in JS-K-mediated TIMP-2 production. In all cell lines, p38 phosphorylation (indicative of activity) was unaffected by the 0.5 μM concentration of JS-K (Figure 6). The 1.0 μM concentration of JS-K decreased p38 phosphorylation by approximately 27%, 62%, and 70% in MDA-MB-231 cells, F10 cells, and MCF-7/COX-2 cells, respectively (Figure 6). At 0.5 and 1.0 μM concentration, JS-K decreased ERK1/2 phosphorylation in F10 cells by 36% and 57%, respectively (Figure 6). In contrast, JS-K did not affect ERK1/2 phosphorylation in MDA-MB-231 cells or MCF-7/COX-2 cells (Figure 6). The phosphorylation of JNK was not affected by JS-K in any cell line (Figure 6).

## Discussion

JS-K is a NO prodrug that releases high levels of NO upon conjugation with glutathione by GST enzymes [15]. JS-K has been shown to inhibit the growth of cancer cells *in vitro *and *in vivo *[15-19]. In addition to its growth inhibitory properties, JS-K induces differentiation in leukemia cells [15] and possesses anti-angiogenic activity *in vitro *[16]. In the present study, we have identified inhibition of breast cancer invasion across the Matrigel basement membrane as another important anticancer activity of JS-K.

Cell invasion involves MMP-mediated proteolysis of the basement membrane, which is counterbalanced by TIMPs. NO donors have been shown to increase and decrease MMP activity, depending on the cell type. NO donor-treated rheumatoid synovial cells increased MMP-2 production, but did not influence the production of TIMP-1 and TIMP-2 [39]. In human fibrosarcoma cells and lung epithelial cancer cells, NO donors inhibited MMP-2 secretion [40].

In the present study, JS-K increased TIMP-2 levels in breast cancer cells. TIMP-2 has been shown to inhibit the invasiveness of breast cancer cells *in vitro *and *in vivo*. Overexpression of TIMP-2 decreased the *in vitro *invasion of ras-transformed breast epithelial cells [41]. Mice injected with TIMP-2-transfected MDA-MB-231 breast cancer cells had a lower number of osteolytic bone metastases and a higher survival rate than mice injected with nontransfected cells [42]. Liposome-complexed *TIMP2 *DNA constructs administered to MMTVneu transgenic mice reduced tumor growth and effectively inhibited the occurrence of lung metastases [43]. Our present findings are consistent with these of TIMP-2 acting as a suppressor of cell invasion. On the other hand, high levels of TIMP-2 have also been correlated with distant metastasis of breast tumors [44,45]. Our data indicate that TIMP-2 is an important mediator of the anti-invasive activity of JS-K. Since inhibition of TIMP-2 did not fully block the anti-invasive effects of JS-K, however, other mechanisms are likely to be involved in the anti-invasive effects of JS-K.

In the present study, JS-K was found to consistently decrease the activity of p38, but not that of ERK1/2 or JNK, in breast cancer cells. p38 has been shown to regulate TIMP-2 expression [37,38]. Downregulation of p38 activity increased TIMP-2 production in squamous cell carcinoma [38]. Phorbol myristate acetate-induced downregulation of TIMP-2 secretion was reversed by inhibition of p38 in glioblastoma cells [36]. p38 activity was decreased only at the higher concentration of JS-K, however, despite the fact that JS-K inhibited the invasiveness of breast cell lines across Matrigel in a dose-dependent manner. p38 is not likely to be the major pathway involved in the anti-invasive activity of JS-K.

## Conclusion

Our results reveal a novel and important function for the NO-releasing prodrug JS-K in suppressing the invasiveness of breast cancer cells across the Matrigel basement membrane. One mechanism by which JS-K inhibits breast cancer cell invasion is the upregulation of TIMP-2 production. The invasion of cancer cells through basement membrane is an essential step in cancer metastasis. The ability of JS-K to suppress this important step in the metastatic process indicates its potential clinical relevance in the chemoprevention and therapy of metastatic breast cancer.

## Abbreviations

COX-2 = cyclooxygenase-2; DMEM = Dulbecco's modified Eagle's medium; ELISA = enzyme-linked immunosorbent assay; ERK1/2 = extracellular signal-regulated kinase 1/2; FBS = fetal bovine serum; GST = glutathione S-transferase; JNK = c-Jun N-terminal kinase; JS-K = O^2^-(2,4-dinitrophenyl) 1- [(4-ethoxycarbonyl)piperazin-1-yl]diazen-1-ium-1,2-diolate; MMP = matrix metalloproteinase; MTS = 3-(4,5-dimethylthiazol-2-yl)-5-(3-carboxymethoxyphenyl)-2-(4-sulfophenyl)-2H-tetrazolium; NO = nitric oxide; NOS = nitric oxide synthase; TIMP = tissue inhibitor of matrix metalloproteinase.

## Competing interests

The authors declare that they have no competing interests.

## Authors' contributions

AMT and A-MS conceived and designed the study. AM-S, VM, and RN-A performed the proliferation assays, invasion assays, NO assays, MMP arrays, ELISAs, and western blot analyses. JES and LKK synthesized JS-K and JS-43-126. MMJ performed the statistical analysis. The manuscript was prepared by AM-S and AMT. All authors read, critically advised, and approved the manuscript.
